# An Evaluation of Effects of Different Mydriatics on Choroidal Thickness by Examining Anterior Chamber Parameters: The Scheimpflug Imaging and Enhanced Depth Imaging-OCT Study

**DOI:** 10.1155/2015/981274

**Published:** 2015-10-05

**Authors:** İsa Yuvacı, Emine Pangal, Sümeyra Yuvacı, Nurettin Bayram, Mustafa Ataş, Burhan Başkan, Süleyman Demircan, Ali Akal

**Affiliations:** ^1^Department of Ophthalmology, Kayseri Training and Research Hospital, 38000 Kayseri, Turkey; ^2^Department of Ophthalmology, School of Medicine, Harran University, 63300 Sanliurfa, Turkey

## Abstract

*Aim*. To assess the effects of mydriatics commonly used in clinical practice on choroidal thickness and anterior chamber change.* Methods*. This was a prospective, randomized, controlled, double-blinded study including a single eye of the participants. The subjects were assigned into 4 groups to receive tropicamide 1%, phenylephrine 2.5%, cyclopentolate 1%, and artificial tears. At the baseline, anterior chamber parameters were assessed using a Pentacam Scheimpflug camera system, and choroidal thickness (CT) was measured using a spectral-domain OCT with Enhanced Depth Imaging (EDI) modality. All measurements were repeated again after drug administration.* Results*. Increases in pupil diameter, volume, and depth of anterior chamber were found to be significant (*p* = 0.000, *p* = 0.000, and *p* = 0.000, resp.), while decreases in the choroidal thickness were found to be significant in subjects receiving mydriatics (*p* < 0.05).* Conclusions*. The study has shown that while cyclopentolate, tropicamide, and phenylephrine cause a decrease in choroidal thickness, they also lead to an increase in the volume and depth of anterior chamber. However, no correlation was detected between anterior chamber parameters and choroidal changes after drug administration. These findings suggest that the mydriatics may affect the choroidal thickness regardless of anterior chamber parameters. This study was registered with trial registration number 2014/357.

## 1. Introduction

Mydriatics have a broad range of applications in ocular examination and treatment to allow for pupil dilatation and/or cycloplegia. In clinical practice, these agents are used to achieve cycloplegia before refraction examination; to achieve pupil dilatation before fundus examination, surgery, and angiography; or to prevent synechia in the management of uveitis. After administration, many changes occur in the iris root, anterior chamber angle, anterior chamber depth, iris thickness, and iris volume as well as pupil dilatation [1–3].

The choroidea is a vascular layer localized in the outer sclera and inner retinal layers. It has many major functions, including oxygenation of inner nuclear layers and retinal pigment epithelium [4]. Greater understanding of the choroidal structure may contribute to the diagnosis and management of several eye disorders. Enhanced Deep Imaging-Optical Coherence Tomography (EDI-OCT) allows a detailed evaluation of choroidea and theoretically contributes to better understanding of a wide range of disorders. In several studies using EDI-OCT, it has been shown that choroidal thickness is affected by many diseases such as age related macular degeneration [5], degenerative myopia [6], and central serous retinopathy [7] and several conditions such as smoking [8], caffeine intake [9], and time of day [10]. There are studies evaluating anterior chamber changes, visual field, and the retinal nerve fiber layer [11, 12].

It is possible that the choroidea, a part of uvea like iris, may display certain changes after mydriatic use. There have been a limited number of studies measuring choroidal thickness after mydriatic use. In these studies, conflicting results were reported after measurements with sufficient dilatation in healthy individuals, suggesting no change [13, 14] or thinning [15] in choroidea by mydriatics. In our study, we have tested the hypothesis that choroidal changes will occur with the administration of 3 commonly used mydriatics and investigated whether these potential changes may be assessed through evaluation of anterior chamber changes.

## 2. Methods

Subjects: this was designed as a randomized, placebo-controlled, double-blinded study including a single eye of participants. The study followed the tenets of the Declaration of Helsinki and was approved by the Local Ethics Committee of Erciyes University (2014/357). All individuals received both oral information and written information about the study, and each was provided with written and informed consent before participation to the study.

All individuals underwent a screening process involving a complete ophthalmologic examination, including visual acuity and refraction, slit-lamp biomicroscopy, fundus examination, and intraocular pressures measured using noncontact tonometry. Axial length was measured using an IOL Master (Carl Zeiss Meditec, Dublin, CA). Anterior chamber parameters were measured using a Pentacam rotating Scheimpflug camera (Oculus, Wetzlar, Germany). The retina nerve fiber layer and choroidal thickness measurements were obtained through the Spectralis OCT (Heidelberg Engineering, Heidelberg, Germany).

All individuals required a best corrected visual acuity of 20/25 or better, with refractive error <5 diopters, 3 diopters of cylinder, and absence of glaucomatous optic disc changes. Exclusion criteria included any retinal diseases, history of ocular injury or surgery, any reasons for poor image quality of OCT such as unstable fixation or severe cataract, and age younger than 18 years.

Two hundred and forty eyes of 120 individuals satisfying the inclusion criteria were enrolled in the study between May 2014 and September 2014. Both eyes were enrolled, and one eye of each individual was randomly selected. A double-blinded protocol was used, and those who evaluated the EDI-OCT did not know which drug was administered to each patient.

All individuals were randomly assigned into 4 groups based on the application of drops. Subjects who received a drop of tropicamide 1% 3 times at 5 min intervals were referred to as the tropicamide group, while subjects who received a drop of 2.5% phenylephrine 3 times at 5 min intervals were the phenylephrine group. Subjects who received a drop of cyclopentolate 1% 3 times at 5 min intervals were defined as the cyclopentolate group, and subjects who received a drop of artificial tears 3 times at 5 min intervals were defined as the control group.

Procedure of image acquisition: the procedure of obtaining EDI-OCT has been previously described [16]. The subfoveal, temporal, and nasal fovea choroidal thicknesses (CTs) were measured by using spectral-domain OCT (Spectralis, wavelength: 870 nm) with EDI modality. Subfoveal, temporal, and nasal fovea CTs were defined as the vertical distance from the hyperreflective line of Bruch's membrane to the hyperreflective line of the inner surface of the sclera. All subjects were imaged by the same experienced retina specialist (İsa Yuvacı). Two independent clinicians (Emine Pangal and Nurettin Bayram) measured CT, and the average of these measurements was used in the analysis. These clinicians did not know about the other's measurements and were also not aware of the group divisions. EDI-OCT images of each subject were obtained before the administration of drops and 50 min after instillation. All scans were performed around the same time of the day, between 11:00 and 12:00, to minimize the possibility of CT changes attributable to diurnal CT fluctuations [17].

Ocular biometrics before and after application of drops in each subject were also assessed with the IOL Master. The measures of axial length (AXL), lens thickness, and anterior chamber depth (ACD) along the visual axis were obtained through a single measurement procedure.

For each subject, corneal thickness, anterior chamber depth (ACD), corneal volume, anterior chamber angle (ACA), corneal curvature, and anterior chamber volume (ACV) were obtained through Pentacam before and after instillation of drops.

Statistical analysis: all data analyses were performed by using the Statistical Package for the Social Sciences (SPSS) for Windows version 22.0 (SPSS Inc., Chicago, IL, USA). The Pearson chi-square test was used to assess qualitative variables. Continuous variables were presented as mean ± standard deviation. Correlation coefficient (CC) was calculated for each of the ocular parameters. The measurement reliability of CC was considered “excellent” if the values of CC > 0.75, “good” if the values of CC > 0.4, and “poor” if the values of CC < 0.4.

For each continuous variable, normality was checked by Kolmogorov-Smirnov test, and it did not depart significantly from a normally distributed sample (*p* > 0.05). Homogeneity of variances was tested by using Levene's test. For parametric statistics, data with normal distribution were analyzed using one-way ANOVA test to compare the groups. When a significant result was obtained, the Tukey test was used for post hoc comparisons. Repeated measurements of anterior chamber parameters, choroidal thickness, and intraocular pressure of the groups were compared by a Paired *t*-test. For nonparametric statistics, data with skewed distribution were analyzed by using Kruskal-Wallis test. When a significant result was obtained, Mann-Whitney *U* test with Bonferroni's correction was used for post hoc comparisons. A *p* value < 0.05 was considered statistically significant.

## 3. Results

Overall, 120 patients (59 males and 61 females) were included in the study. The mean age was 30.9 ± 9.7 years (age range: 18–56 years). All patients were randomly divided into 4 groups. Groups were comprised of the tropicamide group (*n* = 29; 24%), the phenylephrine group (*n* = 29; 24%), the cyclopentolate group (*n* = 32; 27%), and the control group (*n* = 30; 25%). The mean spheric equivalent was measured as −0.55 ± 1.49 diopters in the tropicamide group, −0.73 ± 1.60 diopters in the phenylephrine group, 0.01 ± 2.24 diopters in the cyclopentolate group, and −1.0 ± 1.20 diopters in the control group. There were no significant differences in the demographic characteristics and ocular parameters between those who received mydriatics and those who were in the control group. Demographics and ocular parameters in all groups were summarized in Table 1. Pupil diameter increased from 3.08 ± 0.53 mm at baseline to 6.47 ± 0.69 mm after drug administration in the tropicamide group, from 3.02 ± 0.50 mm to 5.80 ± 0.89 mm in the phenylephrine group, from 3.36 ± 0.72 mm to 6.79 ± 0.74 mm in the cyclopentolate group, and from 3.19 ± 0.52 mm to 3.20 ± 0.51 mm in the control group, respectively. Pupil diameter increased significantly in the subjects who received mydriatics (*p* = 0.000), but the extent of pupil diameter was found to be higher in the tropicamide and the cyclopentolate groups than in the phenylephrine group. The anterior chamber depth increased from 2.88 ± 0.33 mm at baseline to 2.96 ± 0.34 mm after drug administration in the tropicamide group, from 3.03 ± 0.32 mm to 3.11 ± 0.34 mm in the phenylephrine group, from 3.09 ± 0.33 mm to 3.22 ± 0.31 in the cyclopentolate group, and from 3.11 ± 0.34 mm to 3.14 ± 0.38 mm in the control group, respectively.

These results demonstrate that the anterior chamber depth increased significantly in the subjects who received mydriatics when compared to the control group (*p* = 0.000). When it comes to the anterior chamber volume, the study found that it increased from 154.76 ± 34.99 mm^3^ at baseline to 171.90 ± 31.52 mm^3^ after drug administration in the tropicamide group, from 178.28 ± 39.43 mm^3^ to 193.97 ± 34.69 mm^3^ in the phenylephrine group, from 187.93 ± 35.38 mm^3^ to 203.84 ± 30.06 mm^3^ in the cyclopentolate group, and from 184.66 ± 38.97 mm^3^ to 185.10 ± 38.94 mm^3^ in the control group, respectively. These results also show that the anterior chamber volume increased significantly in the subjects who received mydriatics when compared to the control group (*p* = 0.000).

The central EDI-OCT measurements before and after drug administration were 328.65 ± 87.88 *µ* and 302.10 ± 74.29 in the tropicamide group, 341.83 ± 73.36 *µ* and 316.11 ± 68.70 *µ* in the phenylephrine group, 312.28 ± 84.94 *µ* and 292.81 ± 88.69 *µ* in the cyclopentolate group, and 326.53 ± 67.20 *µ* and 316.57 ± 75.51 *µ* in the control group, respectively. These findings indicate that the central EDI-OCT measurements decreased significantly in the subjects who received mydriatics when compared to the control group (*p* = 0.000), but no significant difference was detected among the tropicamide, phenylephrine, and cyclopentolate groups in terms of choroidal thinning. Moreover, no significant difference was also detected in the central choroidal thickness before and after drug administration in the control group (*p* = 0.172).

The temporal EDI-OCT measurements before and after drug administration were 308.90 ± 79.36 *µ* and 291.48 ± 72.80 in the tropicamide group, 324.34 ± 79.36 *µ* and 305.58 ± 66.94 *µ* in the phenylephrine group, 291.41 ± 85.32 *µ* and 280.12 ± 84.30 *µ* in the cyclopentolate group, and 297.47 ± 68.72 *µ* and 305.47 ± 67.87 *µ* in the control group, respectively. Thus, the temporal EDI-OCT measurements saw a significant decrease in the participants who received mydriatics when compared to the participants in the control group (*p* = 0.000). On the other hand, no significant difference was detected in the central choroidal thickness in the control group (*p* = 0.072). The nasal EDI-OCT measurements before and after drug administration were 302.10 ± 84.48 *µ* and 286.03 ± 74.78 in the tropicamide group, 313.45 ± 66.98 *µ* and 296.86 ± 64.30 *µ* in the phenylephrine group, 290.34 ± 85.89 *µ* and 276.87 ± 86.52 *µ* in the cyclopentolate group, and 305.53 ± 81.13 *µ* and 300.77 ± 75.79 *µ* in the control group, respectively. The nasal EDI-OCT measurements significantly decreased in the subjects receiving mydriatics when compared to the control group (*p* = 0.000). No significant differences were detected in the central choroidal thickness in the control group (*p* = 0.232). Changes in the anterior chamber parameters, choroidal thickness, and intraocular pressure before and after drug administration in all groups are summarized in Table 2.

No statistical difference in the choroidal thickness was observed for subjects with an increase in anterior chamber depth and subjects with changes in anterior chamber depth were compared (*r* = −0.136, *p* = 0.200). The same was also true for anterior chamber volume (*r* = 0.071, *p* = 0.505).

In general, the mean for the pupil diameter increased from 3.17 ± 0.85 mm at baseline to 5.57 ± 1.59 mm after drug administration, while the means for the central, subfoveal, and choroidal thicknesses decreased from 326.94 ± 78.59 *µ* at baseline to 308.29 ± 75.55 *µ*. Overall, no statistically significant change was detected between the decrease in the choroidal thickness and the extent of increase in the pupil diameter (PD) (*p* = 0.086). No significant difference was found between the subjects with PD > 6.0 mm and with PD < 6.0 mm in the groups receiving tropicamide 1% and phenylephrine 2.5%, but the extent of the choroidal thinning was found to be higher in the subjects with PD > 6.0 mm in the cyclopentolate group. Overall, no significant correlations were detected between the central choroidal thickness and pupil diameter (*r* = 0.033, *p* = 0.754).

## 4. Discussion

Mydriatics induce pupil dilatation or cycloplegia through several mechanisms, including sympathomimetic and antimuscarinic activities. In Turkey, there are three commercially available mydriatic preparations in common use. Of these, cyclopentolate 1% and tropicamide 1% have antimuscarinic (parasympatholytic) effects. The major difference between these antimuscarinic drugs is the duration of action. Phenylephrine 2.5% is an *α*-agonist with sympathomimetic effects. Generally, sympathomimetic agents have shorter onset and duration of action than antimuscarinic agents. There may be changes in several parameters, such as the iris configuration and angle, the volume and the depth of anterior chamber during pupil dilatation, and cycloplegia induced by mydriatics [1–3]. Length of these changes can cause some mechanical effects on a fixed eye. In addition, the contraction of nonvascular smooth muscle cells innervated by sympathetic and parasympathetic stimuli in choroidea can lead to fluid efflux from choroidea and choroidal thinning by constriction [18–20]. Parasympathetic effects occur in the perivascular plexus, which affects choroidal blood flow and acts as vasodilator [21]. Both drug classes act to increase sympathetic effect. These potential mechanisms suggest that the effects induced by mydriatic agent include choroidal thinning. Theoretically, the mechanical effects of anterior chamber changes induced by drug use, the effect of drugs on choroidea, or both can cause choroidal changes. In the present study, we evaluated pre- and posttreatment choroidal and anterior chamber changes and relationships among these parameters, in order to investigate the effects of mydriatic agents.

In our study, it was found that all mydriatic agents led to significant choroidal thinning. No correlation was demonstrated between choroidal changes and pupil diameter or volume and depth of anterior chamber. In the cyclopentolate group, it was found that the extent of thinning was significantly higher in the subjects with PD > 6,0 mm than those with PD < 6.0 mm. However, no such differences were observed in the tropicamide and phenylephrine groups.

Similarly, Kara et al. [15] found choroidal thinning after administration of 2 different mydriatic agents in their study that measured choroidal thickness. In that study [15], dilatation was performed in one eye, while no drug was administered to the other eye. The authors emphasized that both agents induced choroidal thinning without statistically significant difference between the agents. In that study [15], measurements were performed 60 minutes after drug administration, while choroidal and anterior chamber measurements were repeated 50 minutes after drug administration. However, in our study, we used an additional mydriatic agent, namely, cyclopentolate 1%. Again, the subjects with PD < 6.0 were also included in our study. As a result, choroidal thinning was observed in both drug classes after administration.

Contrary to our results, it was shown that there was no change in choroidal thickness in 2 studies using mydriatic agents [13, 14]. In their study in which choroidal thicknesses were assessed in several quadrants of the central choroidal region before and after Mydrin P (phenylephrine plus tropicamide), Kim et al. [14] reported no significant difference between the results. In their study using tropicamide 1% in healthy and glaucomatous subjects, Mwanza et al. [13] reported that there was no significant difference between the results. In our study, a significant choroidal thickness was detected after drug use. Not only regional differences but also differences in time to measurement after drug administration and active substances used may be a factor of variation for study results. All subjects meeting the eligibility criteria after drug administration were included in our study. Although mydriatics exert similar effects in different individuals, not all individuals have equivalent susceptibility to mydriatics. For example, variation in the extent of increases in pupil diameter comprises a good sample, which is commonly observed in clinical studies. In a study by Kim et al. [14], both parasympatholytic and sympathomimetic effects of drug used in the study may have caused variation in mechanisms acting on choroidea. Manual measurements may have also contributed to these results, which may be completely incidental.

Sander et al. [22] state that according to results of their study in which they examine the anterior chamber and choroidal changes after a single drop mydriatic application there exists no significant statistical difference between pre- and postdrug anterior chamber parameters and choroidal thickness values for placebo and phenylephrine groups. On the contrary, in our study, we observed statistically significant changes in phenylephrine groups, anterior chamber parameters, and choroidal thickness values after mydriatic use. Both individual and/or regional differences and the extent of the penetration of drug into intraocular structures of eyes might be the effective factors for the existence of such different results. This is because, in this study [22], drug application was made as a single drop. Further, the results are not different compared to ones of placebo group. In our study, at least three doses of drug are applied and the measurement is made after 50 minutes. Unlike our study and a few studies dealing with the same issue, in the same study of Sander et al. [22], choroidal thickness is observed for the group where 2% homatropine is used. The author of [22] noted that this observation could be explained by the change in the parasympathetic effect, more dominant in the posterior, owing to drug. Except this [22], there are no studies known about homatropine; however this drug's mechanism of action resembles tropicamide and the effects of both drugs on the anterior chamber are close to each other [3]. Besides different drug usage and individual and/or regional differences, the variation with regard to effective concentration of drug in eyes, aforementioned above, might be explanatory factors for different results.

In conclusion results have failed to demonstrate a correlation between the pupil diameter and choroidal changes in the subjects with PD > 6.0 mm receiving tropicamide 1% and phenylephrine. Only in the group that received cyclopentolate was choroidal thinning found to be significantly different in the subjects with PD > 6.0 mm compared to those with PD < 6.0 mm. The most important difference of cyclopentolate, an antimuscarinic agent, is a longer duration of action than the other agents used. Thus, it may have longer effect in tissue. Given the smaller number of subjects with PD < 6.0 mm, we think it appropriate to reevaluate these results in studies with larger sample size, as these results may be completely incidental.

To the best of our knowledge, ours is the first study comparing anterior chamber changes and choroidal thickness after mydriatic use to be found in the literature. Our results have demonstrated that mydriatic drug use resulted in choroidal thinning, which was found to be independent from both anterior chamber depth and volume.

Although the choroidal effect that resulted from mydriatic use was thinning on average, it was not observed in all participants in the same way. While there was no significant change in some participants, there was an increase in some others. Excluding pupil diameter changes, anterior chamber parameters also displayed individual differences. There was no statistically significant relationship between anterior chamber parameters and choroidal changes. This might lead us to think that there might be some other factors causing changes in individual CT as a result of mydriatic use. This may also explain why there are conflicting findings in the literature. We are in the opinion that this subject should be further analyzed by large-scale works that utilize different types of data.

In the studies on choroidal thickness, it is important to indicate whether results are obtained before or after mydriatic use in order to assess study. Based on these results, it appears to be impossible to determine the extent of drug effects on the choroidea through evaluation of anterior chamber data.

## Figures and Tables

**Table 1:** A summary of demographics and ocular parameters in all groups.

	Tropicamide (*n* = 29)	Phenylephrine (*n* = 29)	Cyclopentolate (*n* = 32)	Control (*n* = 30)	*p* value
Age (year)	32.9 ± 1.4	31.8 ± 1.5	28.9 ± 1.6	30.1 ± 1.4	0.382^a^
Gender (male : female)	17 : 12	16 : 13	14 : 18	12 : 18	0.561^b^
BCVA (Snellen)	1.05 ± 0.12	1.05 ± 0.15	1.04 ± 0.15	1.09 ± 0.15	0.628^a^
SEQ of manifest refraction (diopters)	−0.55 ± 1.49	−0.73 ± 1.60	0.01 ± 2.24	−1.0 ± 1.20	0.117^a^
AXL (mm)	23.4 ± 0.63	23.7 ± 0.81	23.3 ± 0.88	23.5 ± 0.86	0.425^a^

BCVA: Best Corrected Visual Acuity; SEQ: mean spheric equivalent, AXL: mean axial length.

Variables are expressed as mean ± standard deviation; Level of significance *p* < 0.05.

^a^One-way ANOVA test; ^b^Pearson chi-squared test.

**Table 2:** Changes of anterior chamber parameters, choroidal thickness, and intraocular pressure before and after drug administration.

Dilated eye	Tropicamide (*n* = 29)	Phenylephrine (*n* = 29)	Cyclopentolate (*n* = 32)	Control (*n* = 30)
PD (mm)				
Before	3.08 ± 0.53	3.02 ± 0.50	3.36 ± 0.72	3.19 ± 0.52
After	6.47 ± 0.69	5.80 ± 0.89	6.79 ± 0.74	3.20 ± 0.51
Difference	−3.39 ± 0.55	−2.78 ± 0.90	−3.42 ± 0.68	−0.01 ± 0.01
Pa value	0.000	0.000	0.000	0.054
ACD (mm)				
Before	2.88 ± 0.33	3.03 ± 0.32	3.09 ± 0.33	3.11 ± 0.34
After	2.96 ± 0.34	3.11 ± 0.34	3.22 ± 0.31	3.14 ± 0.38
Difference	−0.80 ± 0.05	−0.80 ± 0.08	−0.13 ± 0.09	−0.03 ± 0.17
Pa value	0.000	0.000	0.000	0.290
ACV (mm^3^)				
Before	154.76 ± 34.99	178.28 ± 39.43	187.93 ± 35.38	184.66 ± 38.97
After	171.90 ± 31.52	193.97 ± 34.69	203.84 ± 30.06	185.10 ± 38.94
Difference	−17.14 ± 11.53	−15.69 ± 12.17	−15.91 ± 13.44	−0.44 ± 1.16
Pa value	0.000	0.000	0.000	0.051
CEDİ (*µ*)				
Before	328.65 ± 87.88	341.83 ± 73.36	312.28 ± 84.94	326.53 ± 67.20
After	302.10 ± 74.29	316.11 ± 68.70	292.81 ± 88.69	316.57 ± 75.51
Difference	26.55 ± 33.85	25.72 ± 32.66	19.47 ± 19.41	9.97 ± 38.98
Pa value	0.000	0.000	0.000	0.172
TEDİ (*µ*)				
Before	308.90 ± 79.36	324.34 ± 79.36	291.41 ± 85.32	297.47 ± 68.72
After	291.48 ± 72.80	305.58 ± 66.94	280.12 ± 84.30	305.47 ± 67.87
Difference	17.42 ± 42.70	18.76 ± 38.87	11.29 ± 16.40	−8.00 ± 39.15
Pa value	0.037	0.015	0.000	0.272
NEDİ (*µ*)				
Before	302.10 ± 84.48	313.45 ± 66.98	290.34 ± 85.89	305.53 ± 81.13
After	286.03 ± 74.78	296.86 ± 64.30	276.87 ± 86.52	300.77 ± 75.79
Difference	16.07 ± 40.51	16.59 ± 34.03	13.47 ± 18.06	4.76 ± 21.36
Pa value	0.042	0.014	0.000	0.232
IOP (mmHg)				
Before	14.90 ± 2.94	14.48 ± 2.94	14.97 ± 2.72	14.27 ± 1.53
After	14.34 ± 2.11	14.52 ± 2.10	14.66 ± 2.51	14.10 ± 1.86
Difference	0.55 ± 3.01	−0.40 ± 1.92	0.31 ± 1.59	0.17 ± 1.91
Pa value	0.332	0.924	0.276	0.637

PD: pupil diameter, ACD: anterior chamber depth, ACV: anterior chamber volume, CEDİ: central, subfoveal, choroidal thickness, TEDİ, temporal choroidal thickness, and NEDİ: nasal choroidal thickness.

Variables are expressed as mean ± standard deviation. Level of significance *p* < 0.05, a Paired *t*-test.
